# Quality of life framework in poultry: a methodological approach to characterize the behavior of chickens reared under free-range conditions

**DOI:** 10.3389/fvets.2026.1765083

**Published:** 2026-06-26

**Authors:** Laura Menchetti, Diletta Chiattelli, Cesare Castellini, Angela Trocino, Claudia Ciarelli, Alice Cartoni Mancinelli

**Affiliations:** 1 School of Biosciences and Veterinary Medicine, University of Camerino, Matelica, Italy; 2 Department of Agricultural, Food and Environmental Sciences, University of Perugia, Perugia, Italy; 3 Department of Agronomy Food Natural Resources Animal and Environment, University of Padova, Legnaro, Italy; 4 Department of Agricultural and Food Sciences, Alma Mater Studiorum - University of Bologna, Cesena, Italy

**Keywords:** affect balance, behavior, negative affective states, positive affective states, stereotypy

## Abstract

The “Quality of Life” (QOL) is a relatively recent approach to animal welfare that emphasizes animals’ subjective experiences and the need to ensure long-term positive affective states. In this study, the QOL framework was applied to poultry. Four slow-growing chicken genotypes (A, NN, CB, and LD) reared under free-range conditions were used as a case study to characterize behavioral patterns and gain insights into their welfare and adaptive capacity. Behaviors were monitored over extended observation periods (5 weeks) and classified as positive or negative affects; a quantitative indicator of their balance was then calculated (i.e., proportion of positive to total affects, PPA). The classification of behaviors, such as sheltering and attacking, proved to be challenging, necessitating a careful analysis of their functional significance, occurrence patterns, and distribution across the lifespan of the animal. The PPA index revealed that positive effects occurred more frequently than negative ones across all genotypes, ranging from 73.7 ± 4.8% in LD to 92.5 ± 3.6% in NN, without significant differences among genotypes. The study also highlighted that behavior classification should not be done *a priori* but rather depends on several factors, including occurrence and temporal dynamics, in addition to genotype and rearing conditions. Nonetheless, the QOL approach still presents challenges, and further research is required to refine the classification of behaviors into positive and negative affects and to develop robust indicators of their balance.

## 1 Introduction

In animal science, the link between behavior and welfare is well recognized. If an animal cannot display its species-specific behaviors and expresses abnormal behaviors for a long period, its welfare status is compromised and vice versa (1). This close relationship has stimulated the development of behavioral studies that nowadays range from fundamental ethology to applied behavioral analysis to assess animal adaptability in specific environments or conditions (2, 3), including those for farmed animals. Within this evolving framework, the “Five Freedoms,” while still a cornerstone for assessing animal welfare, have increasingly been regarded as insufficient, as they focus primarily on the absence of suffering while neglecting the role that positive experiences play in determining overall animal welfare (4). According to negative experiences cannot be entirely eliminated; however, they should be minimized to allow greater opportunity for positive experiences. The need to ensure that animals have “a life worth living” is finally being recognized.

In this context, the concept of Quality of Life (QOL) has emerged as a broad framework for evaluating how events experienced by animals during their life affect their overall welfare, primarily through the assessment of their affective states (5). Affective state is an umbrella term that encompasses emotions, which arise from short-term experiences, and moods, which develop from the accumulation of experiences over time (6–8). Specific events can elicit either positive or negative affective states, and the balance between these states determines the level of animal welfare (9). Yeates (10) describe welfare as “a balance of all experiences within a specific period.” Thus, the QOL framework also highlights the importance of the temporal dimension in welfare assessment, conceptualizing quality of life as “welfare over time.” Unlike conventional welfare measures that capture a single moment in time, the QOL approach integrates repeated assessments throughout an animal’s life to provide a cumulative perspective on welfare. Determining whether an animal has experienced “a life worth living,” therefore, depends on long-term observation and the balance between positive and negative experiences across time (11).

Accordingly, the QOL approach requires objective tools to evaluate the balance of affective experiences. In humans, affect balance has been evaluated through instruments, such as the PANAS (Positive and Negative Affect Schedule), which quantify positive and negative affective states, combine them (e.g., by subtraction or ratio), and express the resulting balance on numerical scales (12). However, as these tools rely on self-report questionnaires, their direct translation to animals is inherently limited. Therefore, the key challenge lies in developing reliable approaches to identify and classify animal affective states.

Although far from understanding animal minds, some authors suggest that behavior is the most informative indicator of animals’ affective states (4, 13). According to Rault et al. (14), positive welfare “encompasses animals experiencing positive mental states resulting from rewarding experiences, including having choices and opportunities to actively pursue goals and achieve desired outcomes.” Thus, positive affective states can be identified by behaviors that give animals opportunities to engage in activities that they find rewarding, such as feed acquisition (including foraging, rumination, and hunting), exploration, playing, positive social interactions, and reproductive and parental care behaviors (4, 9, 15). Conversely, based on behavior, negative affective states can be linked to the failure of the manifestation of rewarding behaviors (leading to frustration, displeasure, and distress), to the lack of a sense of control (due to unpredictability or deprivation of essential stimuli such as the freedom of movement), physical discomfort (e.g., hunger, thirst, disease, extreme temperatures, and pain), and emotional discomfort (e.g., fear, anxiety, loneliness, and boredom) (4, 9, 16). Motivation alone, however, is not sufficient to classify behaviors as reflecting positive or negative affect, as the frequency and context of expression should also be considered. For instance, when it occurs at a low rate, gentle feather pecking is considered an important social interaction in poultry and is indicative of a positive affective state (17). Conversely, a high frequency of feather pecking reflects a welfare problem, leading to feather loss and pain (18). Moreover, persistent or stereotyped feather pecking can escalate to cannibalism, potentially resulting in increased mortality. More generally, both the over-expression and under-expression of behaviors, relative to species-typical baselines, can indicate compromised welfare. Behaviors that are excessively repeated or performed at abnormally high frequencies may reflect frustration, stress, or maladaptive coping mechanisms, whereas behaviors that are under-expressed or absent may indicate apathy, reduced motivation, or impaired physical condition (19–21). Therefore, interpreting behavior in terms of affective state requires not only identifying the type of behavior, but also evaluating its level of expression in relation to biologically meaningful norms.

Finally, reactions to stimuli, the perception of experiences, rewarding mechanisms, and the ability to cope with environmental challenges are shaped by both individual traits and species-specific characteristics (14). Consequently, QOL is determined by the subjective experiences of animals (5), which can be influenced by multiple factors, such as housing conditions [e.g., the presence of enrichment, stocking density; (22)] and the animal’s genetic background (23–25).

In poultry production, this has led to increasing attention to the suitability of different genotypes for outdoor rearing systems (23). Several studies have investigated the behavior of different chicken genotypes to address this issue. However, differences in methodology, particularly regarding video analysis and behavior classification, make comparisons among studies difficult (23, 26, 27). This highlights the need for a robust and feasible methodology to characterize the behavior of different chicken genotypes and assess their adaptability to specific rearing conditions.

The aim of this study was to propose a preliminary methodological approach for applying the QOL framework to chickens. To this end, the behavior of different slow-growing chicken genotypes (SG) reared in a free-range system was characterized over a prolonged observation period and used as a case study. Specifically, (i) the observed behaviors were classified into positive and negative affects, considering both their nature and frequency of expression; and (ii) an indicator reflecting the balance between positive and negative affects over time was proposed to illustrate the application of the QOL framework in extensively reared poultry.

## 2 Materials and methods

### 2.1 Animal housing and environmental conditions

The experimental protocol was approved by the Ethical Committee of the University of Perugia (ID: Prot.62705_2020). All methods were performed in accordance with relevant guidelines and regulations (28–30).

The trial was carried out from April to June 2020 in the experimental facilities of the Department of Agricultural, Food and Environmental Sciences of the University of Perugia (Italy). A total of 400 slow-growing one-day-old chicks belonging to four different genotypes (100 chicks/genotype, 50 females and 50 males) were used: two meat-type birds (A and NN; daily weight gain of 30–35 g/day); a dual-purpose chicken (LD, males used primarily for meat production with a daily weight gain of 20–25 g/day, females mainly used for egg production); and a crossbreed between an Italian local breed and a commercial meat type hybrid (CB; daily weight gain of 20–25 g/day).

**Figure 1:**
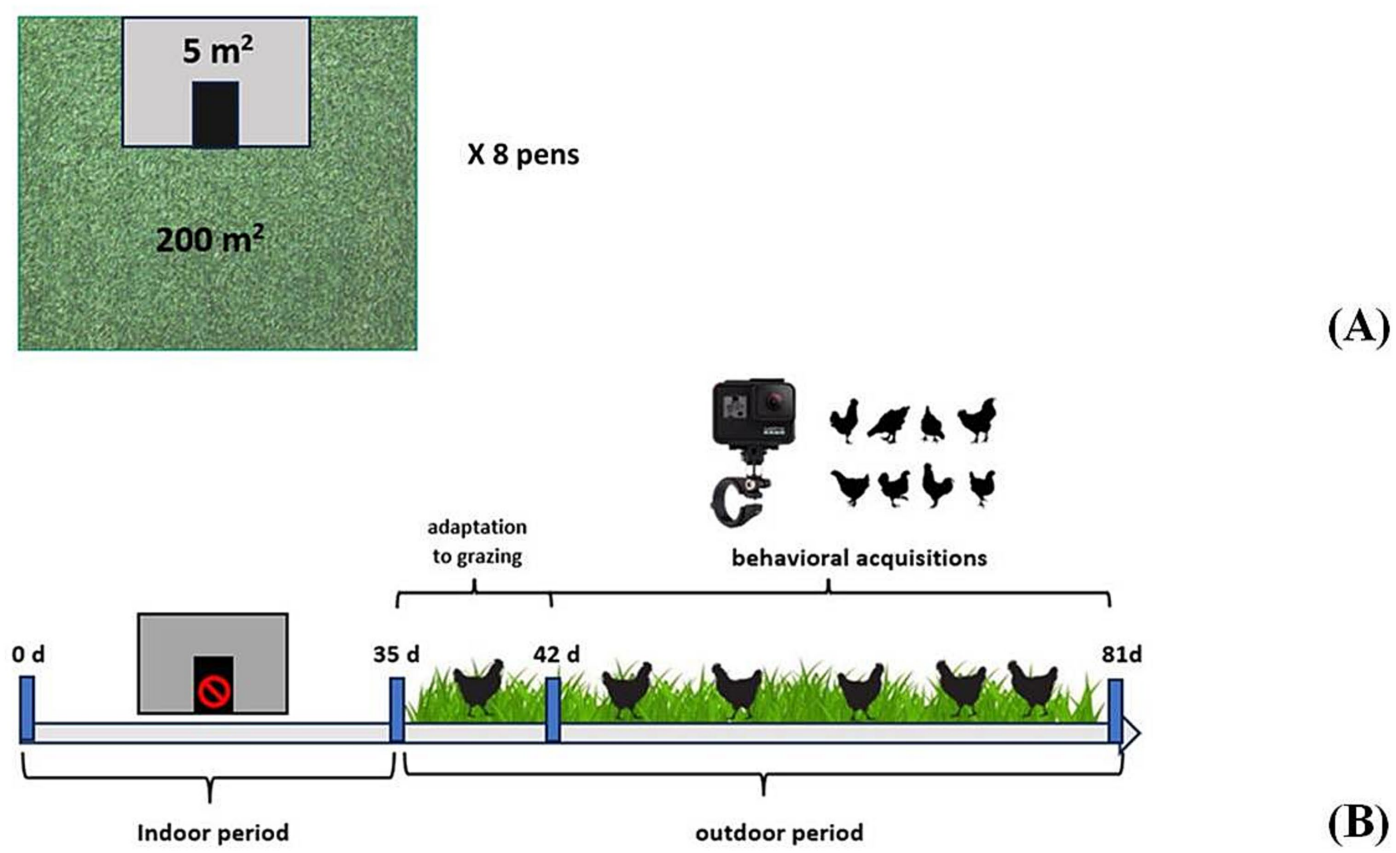
Timeline of the trial **(A)** and graphical representation of the experimental area **(B)**: in gray, the indoor area where chickens were kept until 34 d of age and during the night. In green, the outdoor area used by chickens from 35 days onwards during the daylight.

Each genotype was separately reared; thus, chicks were housed in eight pens (Figure 1A) with access to pasture area (2 pens per genotype; 50 animals each, 25 females and 25 males) with a stocking density of 10 birds/ m^2^ indoor and 4 m^2^ outdoor/bird. The pasture area was covered with spontaneous grass and was similar across the eight pens. All chicks were fed the same commercial diet (starter diet from 1 to 28 d; grower diet from 29 to 81 d) and vaccinated against coccidiosis, Marek, Newcastle infectious bronchitis, and Gumboro disease. Water and feed were provided *ad libitum* for the whole trial. Animals were reared indoors until 35 days under controlled conditions with relative humidity ranging from 65 to 70%; the environmental temperature was 32 °C during the first week, which was then decreased by 2 °C each week until reaching 24–26 °C by 35 days of age. Indoors, all the reported environmental parameters were monitored through the central control units of each pen. After 35 days of age, chickens had free access to the outdoor area during the day until the end of the trial. Access to the outdoor area was guaranteed through the use of automatic doors, which were set to open at 8:00 a.m. and close at 7:00 p.m. At closing time, an operator was always present in order to ensure that all chickens were inside the poultry house. The external temperature was recorded by a thermometer placed outside of the poultry house, and the maximum temperature averaged at 24 °C and the minimum averaged at 16 °C during the day, whereas the minimum was 12 °C during the night when chickens were kept inside the shelter.

### 2.2 Behavioral observations

As described in detail by Cartoni Mancinelli et al. (31), the Noldus Technology (Wageningen, Netherlands) was used to record and analyze animal behaviors. Figure 1B shows the recording timeline where the period 35–42 days of age was considered as the adaptation phase for chickens to the outdoor area. Behavioral observations were carried out from 42 to 81 days of age, corresponding to the end of the rearing period. For each pen, 2-h videos (09:00–11:00 a.m.) per week were recorded, resulting in a total of 10 videos per genotype (five videos per replicate).

Two trained observers analyzed the videos based on the ethogram described in Table 1. Inter-observer agreement, assessed on a subset of observations independently scored by both observers using the Intraclass Correlation Coefficient (ICC), was found to be good (ICC > 0.6 for all behaviors). Each video was analyzed using 10-min sampling intervals (6 scans per hour; 12 scans per video), and 10 s were observed at each scan. Recorded data were expressed as the percentage of animals expressing the behavior out of the total number of visible animals at each scan, and then averaged over the 2-h observation period.

**Table 1:** Description of recorded behaviors of broiler chickens in the outdoor area [modified from (31)].

Behavior	ATOL references^1^	Description
Roosting	ATOL_0000837	Chicken in a lying position with the ventral body region in contact with the floor.
Resting	ATOL_0000816	Chicken with the body in line with the ground, the head is erect and eyes opened. Only the feet are in contact with the floor.
Sleeping	ATOL_0000873	Chicken with the head in a low posture (under the wing or on the litter) and eyes closed.
Walking	ATOL_0000805	Chicken that moves more than three steps.
Running	ATOL_0000806	Chicken that moves more than three steps quickly.
Feed pecking	ATOL_0000363	Chicken that pecks inside the feeder.
Drinking	ATOL_0000361	Chicken that pecks inside drinker.
Other pecking	ATOL_0000845	Chicken that pecks other things.
Self-grooming	ATOL_0000823	Animal preening its own feathers.
Swelling	ATOL_0005361	Chicken puffing out the breast feathers.
Scratching	ATOL_0000360	Chicken that scratches with the paw on the ground.
Stretching	ATOL_0000822	Chicken that stretches the body and legs.
Wings flapping	ATOL_0000822	Chicken that beats its wings with breast protruding and vertically extended posture.
Dust bathing	ATOL_0000824	Chicken that forces the sand or other materials into the plumage by squatting on the ground and making appropriate movements with the body, wings and leg.
Allo-grooming	ATOL_0000826	Chicken that preens the feathers of another conspecific.
Grass pecking	ATOL_0000844	Chicken that pecks the grass.
Attacking	ATOL_0000813	Chicken that fighting with aggression or injurious contact against any conspecific.
Escaping		Chicken that escapes from another conspecific after being attacked.
Sheltering		Chickens run and freeze under the vegetation.

^1^Traits follow the ATOL ontology (https://opendata.inra.fr/ATOL/page/), in accordance with the PILLOW project data management plan.

The behaviors were subsequently classified into categories according to the QOL approach based on the available literature (5, 32–34). In particular, behaviors were grouped into classical ethological categories (e.g., static, active, and ingestive) and into positive or negative affective states, according to their association with reward-related processes, biological function, and welfare outcomes. The initial classification was defined *a priori* based on the literature (5, 32–34). However, to account for the specific rearing conditions and behavioral patterns observed in the dataset, the classification was further refined after inspection of behavioral frequencies and context of expression, particularly to identify potential over- or under-expression of behaviors. Details of this refinement are provided in the Results section.

### 2.3 Statistical analysis

The average percentage of animals engaging in each behavior was calculated as the number of animals performing the behavior at each scan divided by the number of animals visible at that scan, and then averaged across the 2-h observation period for each pen and day. Descriptive statistics were used to present the behaviors as marginal means and standard errors (SE) during the observational period.

To assess the balance between positive and negative affective states, behaviors were first classified according to the QOL approach, and the percentage of positive affects out of the total affective states (PPA Index) was calculated as:

$$\mathrm{PPA}\;\text{Index}=\frac{\text{Positive affects}}{\text{Positive affects}+\text{Negative affects}}$$

Where Positive affect was calculated as the sum of the percentages of animals expressing behaviors categorized as “positive affects” for each pen and day, while negative affect was calculated as the sum of the percentages of animals expressing behaviors categorized as “negative affects” for each pen and day.

The effect of genotype on the recorded behaviors was analyzed using a Generalized Linear Model (GLM). Diagnostic plots were employed to assess data distribution, and the Quasi-Likelihood under Independence Model Criterion (QIC) was used to evaluate model fit. A Tweedie distribution with a log link function was selected for the analysis of individual behaviors and behavioral categories (35). For the variables grass pecking, swelling, and allo-grooming, which showed convergence issues under the Tweedie distribution, alternative models were fitted using a normal distribution with a log link function for Grass pecking and a normal distribution with an identity link function for Swelling. Finally, a gamma distribution with a log link function was applied for the PPA and H Indices. The GLM evaluated the main effect of genotype, with day included as a covariate. The pen was considered the experimental unit and included as the subject variable in the model to account for repeated measurements over time. Sidak corrections were applied for multiple comparisons. In accordance with the QOL approach, only the main effect of genotype is presented in the manuscript, whereas the effect of time is reported in the Supplementary material and expressed as the b coefficient with its SE (Supplementary Table S1).

Behaviors associated with affective states (i.e., included in the positive or negative affective state categories) were further analyzed using a binning technique, which groups continuous data into categorical ranges. In this study, frequencies were divided into three equal ranges based on tertiles, indicating low-, medium-, or high-occurrence behaviors (31, 35). For swelling, allo-grooming, and escaping, only one threshold was applied due to the granularity of the data. The categorization aimed to provide a scale of intensity or duration for these behaviors and to identify anomalous repetition suggestive of stereotypes [i.e., the invariant repetition of a behavioral pattern with no apparent goal or function; (36)].

The association between genotype and frequency categories was assessed using Chi-square tests or Fisher’s exact tests, followed by *z*-tests for post-hoc comparisons. Statistical analyses were performed using SPSS 25.0 (SPSS, an IBM Company, Chicago, IL). Statistical significance occurred when *p* ≤ 0.05.

## 3 Results

### 3.1 Classification of behaviors according to the QoL framework based on literature and observed frequency patterns

**Figure 2:**
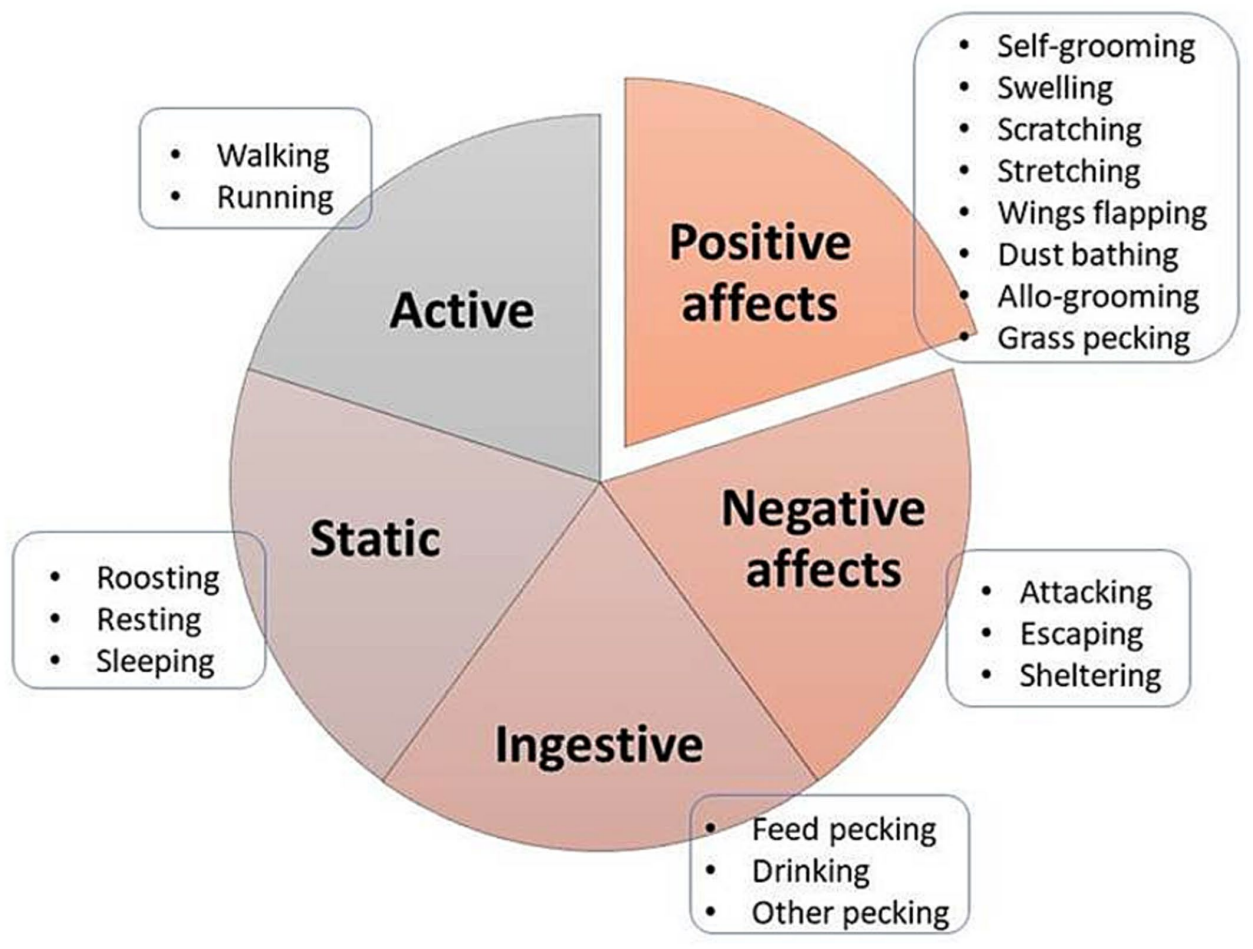
Classification of behaviours into five categories, including positive and negative affective states, based on the available literature and data analysis.

The different behaviors were classified into five categories (static, active, ingestive, and positive and negative affects) (Figure 2). While this classification was primarily grounded in established frameworks (5, 32–34, 37), it was further refined based on the observed frequency and patterns of behavioral expression within the dataset in order to account for potential over- or under-expression under the specific rearing conditions (Supplementary Table S3 with corresponding results). In detail, general maintenance behaviors such as resting, walking, and feed pecking were assigned to the classical behavioral categories, static, active, and ingestive, respectively.

Behaviors that could indicate, cause, or result from animals’ subjective experiential states were classified as either positive or negative affects (5). Positive affects included comfort-related behaviors (i.e., self-grooming, stretching, dust bathing, swelling, and wing flapping), social interactions (i.e., allo-grooming), anticipatory behaviors of reward, and ethotypic consumptive behaviors (i.e., Scratching and Grass pecking) (32, 34, 37). The frequency of expression of these behaviors, although showing differences among genotypes (*p* < 0.05; Supplementary Table S3 and Section 3.3), did not deviate from species-typical patterns to an extent that would justify their classification as over- or under-expressed. They were therefore retained within the positive affects category.

Negative affects encompassed aggressive and defensive behaviors, as well as all behaviors likely to cause injury or fear, such as attacking, escaping, and sheltering (37). Although the practical consequences and affective valence associated with attacking behavior may vary depending on the context, the temporal pattern of occurrence, and the individuals involved, it was conservatively retained within the negative-affect category because of its potential welfare implications (33). Sheltering behavior also warrants further consideration regarding its classification. It was classified as a negative behavior following the evaluation of its frequency and patterns of occurrence, as it was observed at high frequency in certain sessions where it appeared to lack any apparent functional purpose. In particular, the LD genotype showed more than 56% of sessions with a high frequency of occurrence (*p* < 0.05; Supplementary Table S3 and Section 3.2) without an apparent functional explanation (see Discussion section). In addition, it involved a freezing response, a behavior commonly associated with fear (38, 39).

### 3.2 Descriptive analysis of behaviors and behavioral categories

**Figure 3:**
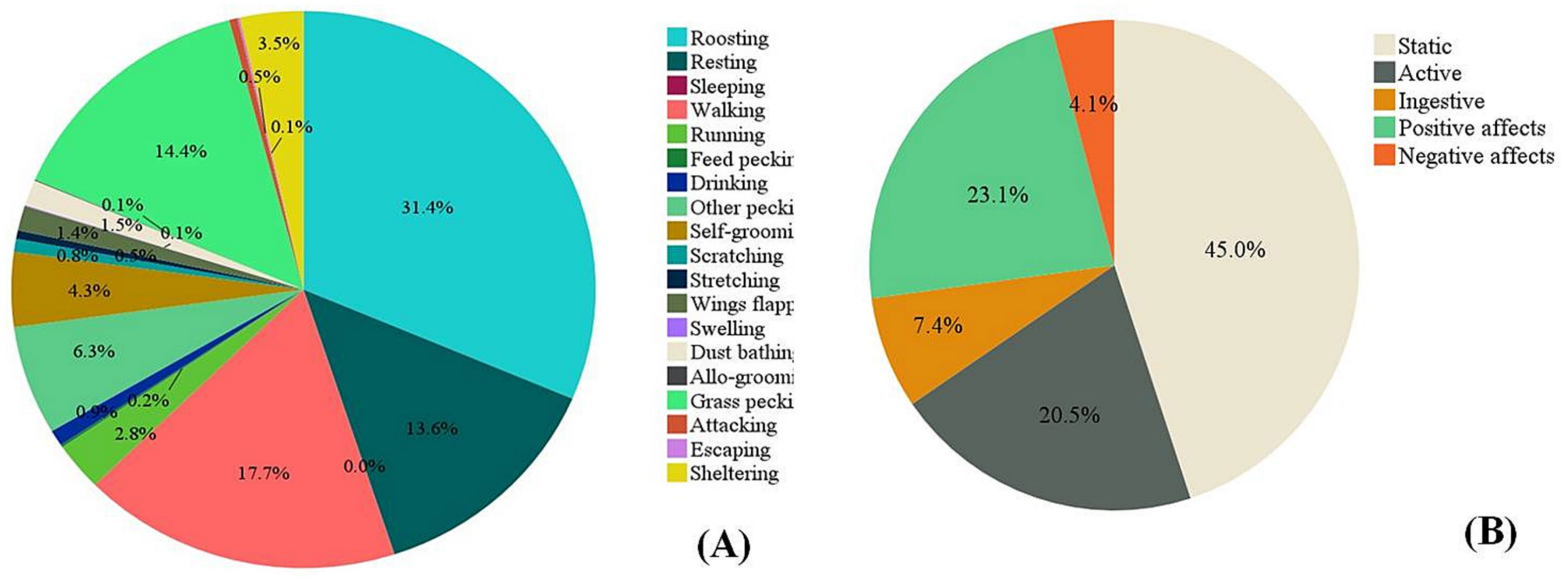
Pie charts showing individual behaviors **(A)** and behaviors grouped into categories **(B)**. Individual behaviors are arranged in clockwise order from the most to the least frequent. Percentages represent the proportion of animals displaying each behavior relative to the total number of visible animals recorded across the entire observation period (10 videos per genotype, each 2 h in duration, from 42 to 81 days of age).

The pie charts display individual behaviors (Figure 3A) and behaviors grouped into categories (Figure 3B) regardless of genotype. Statistical test results are reported in Supplementary Table S1, whereas Supplementary Figure S1 presents the results of pairwise genotype comparisons. Overall, the most represented category was static behaviors (45.0 ± 1.5%), among which the most frequently recorded behavior was roosting (31.4 ± 1.8%). Genotype A showed the highest frequency (*p* < 0.05) of roosting, while genotypes LD and CB showed the lowest frequencies (*p* < 0.001). Across genotypes, the average percentage of resting chickens was 13.6 ± 1.0%, which was higher in CB than in LD (*p* < 0.001).

Active behaviors were recorded at a frequency of 20.5 ± 1.1%, with walking accounting for a mean of 17.7 ± 1.0% of chickens. Genotype A showed the lowest walking frequency (*p* < 0.05). Running occurred at a mean frequency of 2.8 ± 0.4%, with no significant differences among genotypes.

The ingestive behavior category accounted for 7.4 ± 0.7%, and only “other pecking” differed among groups, with LD and CB exhibiting the highest values (Supplementary Figure S1, *p* < 0.05).

The frequency of behaviors classified within the positive affect category was 23.1 ± 0.8%. The most frequently observed behavior was grass pecking (14.4 ± 0.8%), followed by self-grooming (4.3 ± 0.5%), dust bathing (1.5 ± 0.5%), wing flapping (1.4 ± 0.2%), and stretching (0.5 ± 0.1%). Allo-grooming (0.1 ± 0.1%) and swelling (0.1 ± 0.1%) were rare, observed in less than 0.5% of animals. Significant differences among genotypes were observed for grass pecking, dust bathing, scratching (Supplementary Figure S1, *p* < 0.001), and allo-grooming (*p* < 0.05). Grass pecking was more frequent in the NN genotype (17.3 ± 1.8%) compared to LD (11.3 ± 3.6%; *p* = 0.046), whereas LD showed the highest percentages of dust bathing and scratching (*p* < 0.001). Finally, allo-grooming was more frequently expressed in genotype A (0.12 ± 0.03) compared to CB and NN (0.00 ± 0.03; *p* = 0.012).

**Figure 4:**
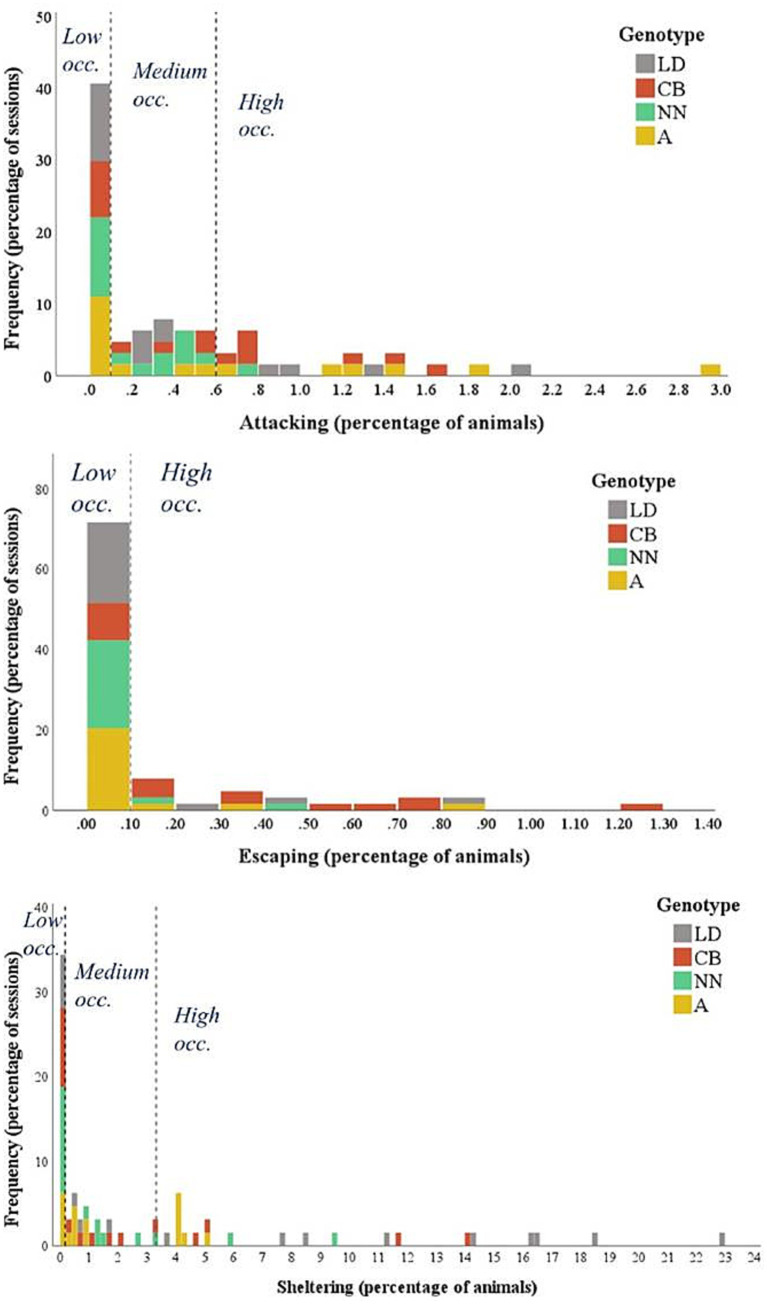
Histograms showing the distribution of occurrences for behaviors in the negative affects category (attacking, escaping, and sheltering) by genotype. In each session, behaviors indicative of negative affects were classified into categories reflecting low, medium, or high occurrence (the dashed lines indicate the thresholds identified for this classification).

The negative affect category accounted for 4.1 ± 0.7% of the total. Aggressive attacks and escaping behaviors were exhibited by 0.5 ± 0.1% and 0.1 ± 0.1% of animals, respectively, with no significant differences among genotypes. The percentage of animals sheltering in vegetation was 3.5%, but it varied considerably among genotypes. In some sessions, more than 20% of LD chickens were observed sheltering, whereas this behavior never exceeded 10% in NN and A chickens. On average, sheltering occurred in nearly 8% of observations for LD chickens compared to less than 3% for the other genotypes (Figure 4, *p* < 0.05). Further analyses of genotype differences across the various behavioral categories are provided in the Supplementary Figure S2 and accompanying text. Regarding temporal changes, a reduction was observed in walking, running, grass pecking, wing flapping, and attacking behaviors, whereas roosting increased over time (*p* < 0.05; Supplementary Table S1).

### 3.3 Focus on positive and negative affects and PPA index

**Figure 5:**
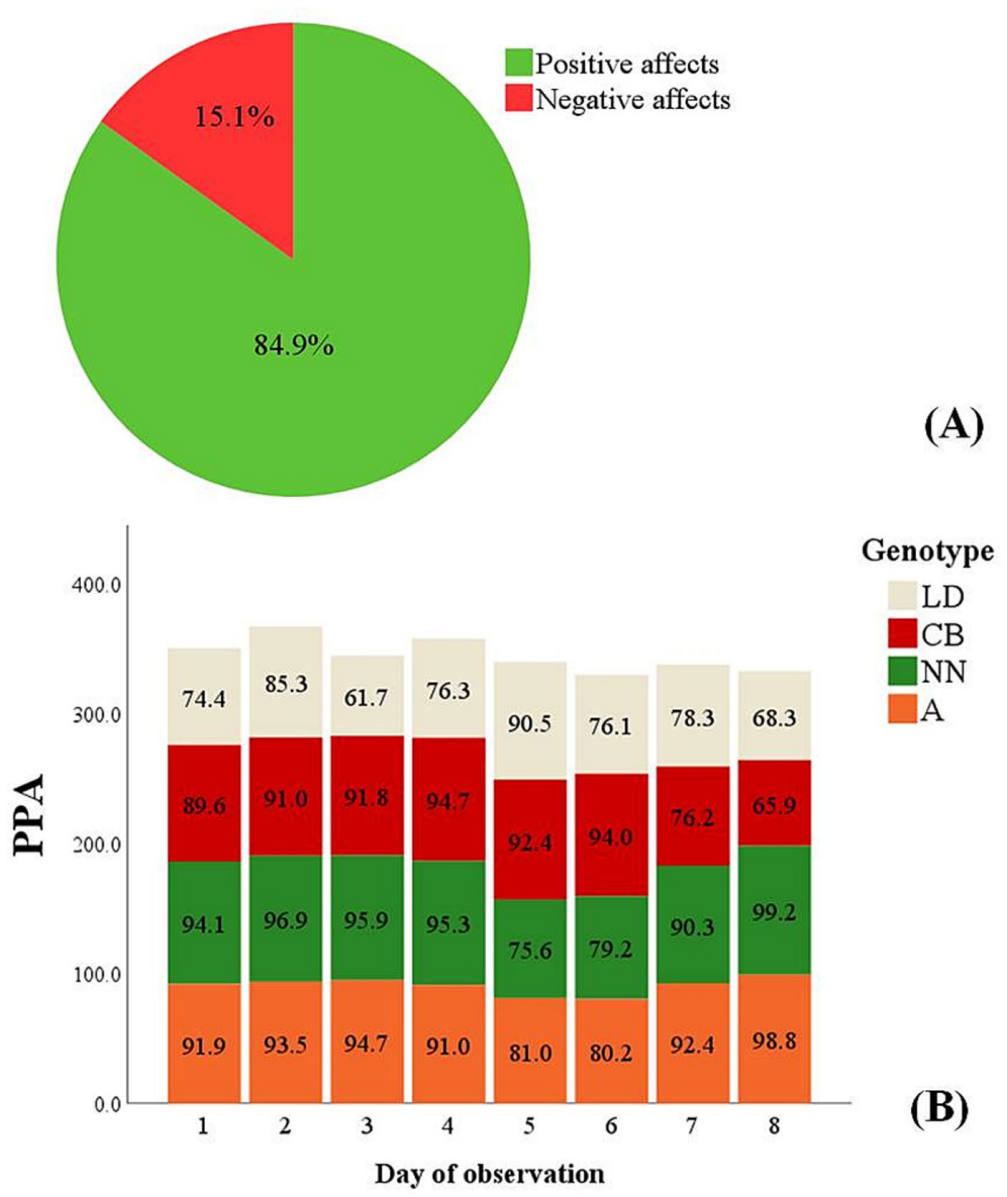
Pie charts illustrating the proportion of positive and negative affect over the entire observation period **(A)**, and the percentage of positive affect relative to total affective states (PPA Index) across genotypes and observation days **(B)**.

Overall, positive affective states were approximately fivefold more frequent than negative ones (Figure 5A). The proportion of positive affect relative to total affective states (PPA index) averaged 86.1 ± 1.9%, ranging from 73.7 ± 4.8% in LD to 92.5 ± 3.6% in NN. No significant differences were observed across days of observation (*p* = 0.105) or genotypes (*p* = 0.127; Figure 5B).

Finally, to account for the frequency of occurrence of behaviors indicative of affective states (and to capture, for instance, potential over-manifestations), these behaviors were categorized in each session as low-, medium-, or high-occurrence behaviors using the binning technique (Supplementary Tables S2, S3). Regarding positive behaviors, CB and A showed a higher proportion of sessions with a high occurrence of self-grooming compared to NN, while both CB and LD had a higher proportion of sessions with high occurrences of scratching and dust bathing. Sessions with a high occurrence of allo-grooming were observed only in LD and A. Finally, NN showed the highest proportion of sessions with a high occurrence of grass pecking (Supplementary Table S3; for all: *p* < 0.05).

Regarding negative behaviors, CB exhibited a greater proportion of sessions with a high occurrence of attacking and escaping compared to NN (*p* < 0.05). In addition, LD showed the highest proportion of high-frequency sheltering observations (Figure 4 and Supplementary Table S3; *p* < 0.05).

## 4 Discussion

To our knowledge, this is the first study in which the QOL framework has been applied to characterize the behavior of chicken genotypes reared in a free-range system. This approach highlights the importance of affective states in evaluating animal welfare, but it also emphasizes the challenges of translating, *a priori*, the conventional categories of an ethogram (e.g., active, ingestive, or comfort behaviors) into positive or negative affects, as proposed by the QOL framework. In this study, behavioral classification primarily relied on the concept of reward to identify positive affects, whereas the failure to manifest rewarding behaviors, discomfort-related behaviors, and behaviors likely to result in negative physical consequences (e.g., injury) were interpreted as indicators of negative affects. Moreover, behavioral frequency was also considered to identify over- or under-expressed behaviors that may become non-functional or even stereotyped under certain conditions, and may therefore be interpreted as indicators of negative affective states (19–21). Consequently, within the QOL framework, behavioral classification should be dynamic and may vary according to genotype, rearing environment, and behavioral expression patterns. This study also proposes a simple ratio of positive to total affects as an indicator of balance over time.

As a case study to explore the application of the QOL framework to poultry, four slow-growing (SG) chicken genotypes were reared in a free-range system and monitored over a 5-week period to estimate their “cumulative welfare” (40). Although based on a limited number of replicates, this experimental design allowed different SG genotypes to be tested under the same conditions (i.e., free-range systems), thereby enabling the recording and classification of a wide range of behaviors.

The most frequently expressed behavior among the genotypes studied was roosting, followed by walking and grass pecking. Grass pecking, a rewarding activity classified as a positive affect, can only be expressed when pasture is available, thereby conferring intrinsic welfare advantages to organic and free-range production systems. The outdoor area functions as environmental enrichment, providing animals with meaningful opportunities for choice. This, in turn, supports the expression of species-specific behaviors (23, 34, 41), thereby contributing to an improved QOL. In our study, the high frequency of walking and grass pecking may suggest that chickens used walking as a purposeful strategy to access and consume grass. This hypothesis is supported by the pattern expressed by genotypes NN and A, which likely showed a positive interaction with the environmental complexity of the pasture system through the occurrence of the above-mentioned behaviors. In our previous study (42), we investigated the motivation of laying hens to access pasture and compared the behavior of indoor and outdoor hens. Motivation was quantified by determining the cost that hens were willing to pay to gain access to the pasture, using a door leading to the outdoor area with progressively increasing weights. Results showed that hens were highly motivated to access the outdoor area, where they could find grass and other valuable resources and express species-specific behaviors. In particular, outdoor hens exhibited a higher frequency of walking and self-grooming and a lower frequency of resting behaviors compared with indoor hens. Overall, these findings support the view that grass pecking is a highly motivated behavior and should be considered an indicator of positive affect.

Consistent with these findings, genotype A also showed a high occurrence of allo-grooming and high levels of self-grooming in multiple observation sessions. These behaviors were classified as a positive affective state, further supporting the positive interaction of these genotypes with the outdoor area (5, 32).

Based on the available literature, additional behaviors classified as positive affective states included scratching and dust bathing (33, 43). In particular, scratching is considered the appetitive component of feeding behavior (43), while dust bathing is so strongly motivated that EFSA defines it as a “high priority behavior” (33). The intrinsic motivation behind these innate behaviors is evident from their expression even in the absence of appropriate substrates (16, 44), and both are typically classified as comfort behaviors in welfare assessment protocols (37). By providing greater behavioral freedom, the free-range system enhances the expression of such positive welfare indicators (8, 45).

In this study, differences in behaviors associated with positive affective states among genotypes may partly reflect their different selection goals. Artificial selection is known to influence the frequency of behavioral expression without altering the animal’s behavioral repertoire (46, 47). In meat-type chickens, selection for high growth rate and production efficiency has been associated with reduced locomotor activity and a lower expression of energetically costly active behaviors, such as dust bathing and exploration (48), likely because a greater proportion of dietary energy is diverted toward body mass accretion rather than other physiological functions (49). Accordingly, the LD genotype, a dual-purpose breed and the lightest genotype in this study, showed a higher frequency of scratching and dust-bathing behaviors, which are innate activities typically associated with laying hens (33, 37). Similarly, the CB genotype, the least selected and lacking a clearly defined productive orientation, exhibited higher levels of scratching and self-grooming. In contrast, although the NN and A genotypes are classified as slow-growing, they are still selected for meat production and displayed higher frequencies of self- and allogrooming behaviors, which are commonly reported in broiler chickens.

Attacking and escaping, indicative of aggressive, threatening, or defensive states, were classified as negative affects. All genotypes in this study displayed low frequencies of these behaviors, confirming the absence of physical aggression throughout the rearing cycle. However, the higher proportion of sessions in which attacking behavior was frequently observed in genotype CB enabled us to make several considerations. As previously mentioned, this pattern could be attributed to the lower degree of selection in CB. Given that behavioral engagement entails an energetic cost (50), it is plausible that intensive selection for productivity has progressively reduced the expression of such behaviors in highly selected hybrids. In contrast, these traits appear to have been retained in the less selected breeds (51). This highlights the importance of including different SG genotypes, even the less-selected ones, within the same experimental design to capture differences in behavioral frequency and temporal distribution.

Importantly, the frequency of aggressive behavior decreased over time, suggesting that these episodes mainly occurred during the early phases of social structuring, when such behaviors help establish group hierarchy (52, 53). Given the central role of hierarchical organization in chicken social structures, where aggressive and escape behaviors serve to establish, rather than disrupt, social rank, and the recognized positive effects of a stable hierarchy on group welfare (53, 54), classifying these behaviors unilaterally as “negative” may be misleading.

Nevertheless, the classification of attacking behavior as unilaterally negative deserves further consideration. Although aggressive interactions are commonly interpreted as indicators of negative affect, the affective state experienced by the individuals involved may depend on the context and outcome of the interaction. In particular, dominance-related behaviors may reflect perceived control or successful social competition, potentially being associated with non-negative or even positive affective states in the attacking individual (55). In this perspective, the affective valence of aggressive encounters may differ between opponents, with negative affect being more likely experienced by the losing or injured individual. However, social fighting is also associated with fear- and anger-like emotional states and may result in injury or distress in both opponents (56). Therefore, given its potential welfare implications, attacking behavior was conservatively retained within the category of negative-affect indicators, in line with the EFSA scientific opinion (33).

The functional significance of behaviors should therefore also be considered when classifying them into affective categories, taking into account not only their qualitative nature but also their frequency and temporal dynamics. This was particularly evident in the case of Sheltering behavior, whose classification was challenging and required consideration of its underlying motivation, genetic background, and frequency of expression. In the LD genotype, sheltering behavior accounted for more than 20% of the observations in some sessions and exceeded the high-occurrence threshold in over 55% of cases, with both occurrence and frequency markedly higher than in the other genotypes. The motivation for this behavior may include the use of natural cover (e.g., trees, bushes) as an anti-predator strategy or as a means to mitigate heat stress (57), behaviors that are not necessarily indicative of negative affective states but may represent adaptive responses. However, in the present study, no predators were present, and the trial was not conducted during the warmest season. Another hypothesis is that, in LD birds, a dual-purpose genotype, sheltering may partly reflect an intrinsic tendency toward brooding, which could influence general behavior and increase the need for a safe resting place. Nevertheless, brooding motivation has been reported to emerge after 81 days of age (58), whereas birds in this study were slaughtered earlier. Therefore, the overexpression of sheltering behavior appeared to lack an obvious functional purpose and may indicate limited confidence in the outdoor area or, in some cases, a stereotyped component. Finally, sheltering involved a freezing response, a behavior commonly associated with fear in many animal species, including poultry (38, 39). Based on these considerations, sheltering was classified as a negative behavior, although some uncertainty remains. This case highlights the challenge of unequivocally assigning specific actions to positive or negative affective states.

The PPA index developed in this study draws inspiration from indices commonly used in human studies (12) and aims to provide a numerical quantification of the balance between negative and positive affects, a concept central to the QOL framework. The PPA indicated a clear predominance of positive affects over negative ones in all genotypes. In addition, the index could quantify this prevalence, showing that positive affects accounted for approximately 74% of all affective states in LD and 93% in NN.

In addition to evaluating the balance between positive and negative affects, analyzing the frequency of these behaviors may help identify environmental and intrinsic determinants of affective states and offer practical insights for improving on-farm welfare management. A high frequency of behaviors associated with negative affective states could indicate the need for corrective actions, whether through improved management practices, resource provisions, or the selection of a genotype better suited to the rearing system. For instance, the high frequency of sheltering behavior in LD could suggest that environmental adjustments are required. Enriching the outdoor area with natural covers, like trees and shelters, may help reduce perceived predation risk and improve behavioral confidence.

Nonetheless, several methodological challenges still remain. The classification of behaviors into positive and negative affective states, based on both their nature and frequency, and the concurrent development of indicators to express their balance, still lacks the methodological rigor needed to detect subtle differences. The development of the PPA index represents a preliminary step toward quantifying the balance between positive and negative affects over time; however, further refinement and validation are required. A broader discussion within the scientific community on the classification of behaviors into positive and negative affects is warranted (14). A consensus-building approach, such as the Delphi method, could help validate or challenge the preliminary framework proposed in this study. In addition, integrating multiple complementary strategies could further validate both the behavioral classification and the results of the PPA index. These approaches may include the use of physiological indicators of stress and welfare, preference and motivation tests to assess the subjective value of behavioral outcomes, and experimental designs in which environmental conditions are manipulated to observe consistent changes in behavioral expression. Altogether, combining expert consensus with empirical validation across behavioral, physiological, and functional domains may provide a more robust and widely accepted framework for the practical application of the QoL approach. This study was also limited by the small number of replicates per genotype. However, the objective of the study was not to perform direct genotype comparisons. Rather, different genotypes were selected to capture a broad repertoire of chicken behaviors and to explore a possible application of the QOL framework.

To our knowledge, this is the first study to apply the QOL concept in chickens raised in free range conditions, highlighting its potential as a promising tool to evaluate genotype characteristics and genotype–environment interaction, although further progress is needed before its routine application. This approach emphasizes the expression of behaviors that contribute to negative or positive effects throughout the animal’s life and, in the present study, revealed distinct repertoires among four SG genotypes. While the proportion of positive affects exceeded that of negative ones across all genotypes, each displayed a particular behavioral profile. These differences may be influenced by the productive purpose of the genotype (dual-purpose vs. meat-type). Although classifying behaviors into positive and negative affective states remains challenging, this preliminary study emphasizes that behaviors cannot be categorized *a priori* and that classification should account for both their frequency and temporal distribution. For example, the temporal distribution of attacking may suggest a shift from a negative to a positive affective state associated with the establishment of a stable social structure. Moreover, the affective valence associated with attacking behavior itself may differ between opponents, as dominance-related interactions could reflect perceived control or successful social competition, potentially corresponding to a positive affective state in the attacking individual, whereas the negative affective component may be primarily associated with the losing or injured subject. With regard to sheltering behavior, its high frequency, particularly in one genotype, led us to classify it as an indicator of negative affect.

In conclusion, in the present study, the classification of behaviors into positive or negative affective states was based on multiple lines of evidence, including the concept of reward, the functional consequences of behaviors (e.g., associations with injury, stress, or social interactions), their frequency, and their contexts of expression. Nevertheless, important challenges and limitations remain, and further studies are needed to standardize the classification of behaviors into affective categories and to develop robust indicators capable of capturing their dynamic balance. In addition, the application of this approach should be extended beyond the present case study to test its robustness across different genotypes, management conditions, and production systems. Advancing these tools will be essential for integrating QOL assessment into routine welfare monitoring and genotype evaluation in alternative poultry systems.

## Data Availability

The raw data supporting the conclusions of this article will be made available by the authors, without undue reservation.
