# Supplementary material for: Quality of life framework in poultry: a methodological approach to characterize the behavior of chickens reared under free-range conditions
Source: Front Vet Sci. 2026 Jun 26;13:1765083. doi: 10.3389/fvets.2026.1765083 (PMC13349751; doi:10.3389/fvets.2026.1765083)
Supplement: Supplementary file 1 [file Table_1.DOCX]

***Supplementary material***

**Table 1 SM.** Results of Generalized Linear Model evaluating the effect of genotype (df = 3) with day included as a continuous covariate (df = 1).

| **Behaviour** | **B coefficient ± standard error for time effect** | **Genotype** | | **Day** | |
| --- | --- | --- | --- | --- | --- |
|  |  | **Wald Chi-Square** | **P value** | **Wald Chi-Square** | **P value** |
| **Roosting** | 0.072±0.021 | 17.457 | **<0.001** | 11.127 | **<0.001** |
| **Resting** | -0.012±0.029 | 8.057 | **0.045** | 0.162 | 0.687 |
| **Sleeping** | - |  | - |  | - |
| **Walking** | -0.041±0.018 | 36.213 | **<0.001** | 5.022 | **0.025** |
| **Running** | -0.337±0.046 | 1.430 | 0.699 | 37.283 | **<0.001** |
| **Sheltering** | 0.120±0.073 | 18.654 | **<0.001** | 2.723 | 0.099 |
| **Feed pecking** | 0.008±0.213 | 0.756 | 0.384 | 0.001 | 0.972 |
| **Drinking** | 0.197±0.121 | 11.015 | **0.012** | 2.626 | 0.105 |
| **Grass pecking** | -0.050±0.021 | 10.624 | **0.014** | 5.592 | **0.018** |
| **Other pecking** | -0.031±0.028 | 15.860 | **<0.001** | 0.484 | 0.487 |
| **Self-grooming** | 0.075±0.045 | 6.183 | 0.103 | 2.320 | 0.128 |
| **Scratching** | -0.095±0.084 | 28.446 | **<0.001** | 1.274 | 0.259 |
| **Stretching** | 0.133±0.040 | 5.627 | 0.131 | 2.154 | 0.142 |
| **Wings flapping** | -0.293±0.072 | 4.465 | 0.215 | 11.179 | **0.001** |
| **Swelling** | 0.010±0.011 | 3.121 | 0.373 | 0.916 | 0.339 |
| **Dust bathing** | 0.053±0.11 | 31.623 | **<0.001** | 0.276 | 0.599 |
| **Attacking** | -0.168±0.062 | 3.283 | 0.350 | 4.854 | **0.028** |
| **Escaping** | -0.201±0.141 | 7.599 | 0.055 | 2.033 | 0.154 |
| **Allo-grooming** | -0.011±0.008 | 10.008 | **0.018** | 1.952 | 0.162 |
| **Others** | - |  | - |  | - |

p values in bold indicate statistical significance for the genotype effect (p<0.05)

**Table 2SM**. Occurrence intervals, defined by lower and upper limits, for the Low-, Medium-, and High-occurrence categories of positive and negative behaviors.

| **Behaviour** | **Occurrence Category** | **Lower threshold** | **Upper threshold** |
| --- | --- | --- | --- |
| **Self-grooming (Binned)** | **Low** | 0.00 | 2.30 |
|  | **Medium** | 2.37 | 4.53 |
|  | **High** | 4.58 | 15.03 |
| **Scratching (Binned)** | **Low** | 0.00 | 0.00 |
|  | **Medium** | 0.12 | 0.53 |
|  | **High** | 0.73 | 7.11 |
| **Stretching (Binned)** | **Low** | 0.00 | 0.00 |
|  | **Medium** | 0.13 | 0.38 |
|  | **High** | 0.41 | 4.27 |
| **Wings flapping (Binned)** | **Low** | 0.00 | 0.00 |
|  | **Medium** | 0.24 | 1.39 |
|  | **High** | 1.43 | 8.73 |
| **Swelling (Binned)** | **Low** | 0.00 | 0.00 |
|  | **High** | 0.17 | 1.39 |
| **Dust bathing (Binned)** | **Low** | 0.00 | 0.00 |
|  | **Medium** | 0.18 | 0.43 |
|  | **High** | 0.55 | 25.04 |
| **Allo-grooming (Binned)** | **Low** | 0.00 | 0.00 |
|  | **High** | 0.17 | 0.83 |
| **Grass pecking (Binned)** | **Low** | 0.22 | 12.77 |
|  | **Medium** | 12.89 | 16.85 |
|  | **High** | 17.17 | 26.33 |
| **Attacking (Binned)** | **Low** | 0.00 | 0.00 |
|  | **Medium** | 0.08 | 0.49 |
|  | **High** | 0.56 | 2.94 |
| **Escaping (Binned)** | **Low** | 0.00 | 0.00 |
|  | **High** | 0.10 | 1.25 |
| **Sheltering (Binned)** | **Low** | 0.00 | 0.17 |
|  | **Medium** | 0.18 | 3.29 |
|  | **High** | 3.33 | 22.92 |

**Table 3SM.** Cross-tabulation of the percentage of behaviors (within Genotype) classified into Low-, Medium-, and High-occurrence categories across the different genotypes. Pearson’s chi-square values and corresponding p-values are reported (df=6), and results of pairwise comparisons are based on z-tests.

| **Classification** | **Behaviour (Binned)** | **Category** | **Genotype** | | | | **Total** | **Pearson Chi-square** | **p-value** |
| --- | --- | --- | --- | --- | --- | --- | --- | --- | --- |
|  |  |  | **LD** | **CB** | **NN** | **A** |  |  |  |
| **Negative** | **Attacking** | **Low** | 43.8%_a_ | 31.3%_a_ | 37.5%_a_ | 43.8%_a_ | 39.1% | 11.536 | 0.073 |
|  |  | **Medium** | 31.3%_a,b,c_ | 12.5%_c_ | 50.0%_b_ | 12.5%_a, c_ | 26.6% |  |  |
|  |  | **High** | 25.0%_a,b,c_ | 56.3%_c_ | 12.5%_b_ | 43.8%_a, c_ | 34.4% |  |  |
|  | **Escaping** | **Low** | 81.3%_a_ | 37.5%_b_ | 87.5%_a_ | 81.3%_a_ | 71.9% | 12.676 | 0.007 |
|  |  | **High** | 18.8%_a_ | 62.5%_b_ | 12.5%_a_ | 18.8%_a_ | 28.1% |  |  |
|  | **Sheltering** | **Low** | 25.0%_a_ | 31.3%_a_ | 50.0%_a_ | 25.0%_a_ | 32.8% | 7.532 | 0.289 |
|  |  | **Medium** | 18.8%_a_ | 43.8%_a_ | 31.3%_a_ | 37.5%_a_ | 32.8% |  |  |
|  |  | **High** | 56.3%_a_ | 25.0%_a, b_ | 18.8%_b_ | 37.5%_a, b_ | 34.4% |  |  |
| **Positive** | **Self-grooming** | **Low** | 37.5%_a_ | 18.8%_a_ | 50.0%_a_ | 25.0%_a_ | 32.8% | 7.688 | 0.262 |
|  |  | **Medium** | 37.5%_a_ | 37.5%_a_ | 37.5%_a_ | 25.0%_a_ | 34.4% |  |  |
|  |  | **High** | 25.0%_a,b_ | 43.8%_a_ | 12.5%_b_ | 50.0%_a_ | 32.8% |  |  |
|  | **Scratching** | **Low** | 12.5%_a_ | 18.8%_a_ | 68.8%_b_ | 75.0%_b_ | 43.8% | 23.257 | <0.001 |
|  |  | **Medium** | 31.3%_a_ | 25.0%_a_ | 18.8%_a_ | 18.8%_a_ | 23.4% |  |  |
|  |  | **High** | 56.3%_a_ | 56.3%_a_ | 12.5%_b_ | 6.3%_b_ | 32.8% |  |  |
|  | **Stretching** | **Low** | 43.8%_a,b, c_ | 75.0%_c_ | 25.0%_b_ | 62.5%_a, c_ | 51.6% | 11.758 | 0.067 |
|  |  | **Medium** | 25.0%_a_ | 0.0%_b_ | 25.0%_a_ | 6.3%_a, b_ | 14.1% |  |  |
|  |  | **High** | 31.3%_a_ | 25.0%_a_ | 50.0%_a_ | 31.3%_a_ | 34.4% |  |  |
|  | **Wings flapping** | **Low** | 31.3%_a_ | 68.8%_b_ | 18.8%_a_ | 18.8%_a_ | 34.4% | 12.294 | 0.055 |
|  |  | **Medium** | 37.5%_a, b_ | 12.5%_b_ | 43.8%_a_ | 37.5%_a, b_ | 32.8% |  |  |
|  |  | **High** | 31.3%_a_ | 18.8%_a_ | 37.5%_a_ | 43.8%_a_ | 32.8% |  |  |
|  | **Swelling** | **Low** | 93.8%_a_ | 87.5%_a_ | 100.0%_a_ | 81.3%_a_ | 90.6% | 3.678 | 0.490 |
|  |  | **High** | 6.3%_a_ | 12.5%_a_ | 0.0%_a_ | 18.8%_a_ | 9.4% |  |  |
|  | **Dust bathing** | **Low** | 25.0%_a_ | 62.5%_b_ | 43.8%_a, b_ | 75.0%_b_ | 51.6% | 14.293 | 0.025 |
|  |  | **Medium** | 18.8%_a, b_ | 0.0%_b_ | 25.0%_a_ | 18.8%_a, b_ | 15.6% |  |  |
|  |  | **High** | 56.3%_a_ | 37.5%_a_ | 31.3%_a, b_ | 6.3%_b_ | 32.8% |  |  |
|  | **Allo-grooming** | **Low** | 81.3%_a, b_ | 100.0%_b_ | 100.0%_b_ | 62.5%_a_ | 85.9% | 12.800 | 0.004 |
|  |  | **Medium** | 18.8%_a, b_ | 0.0%_b_ | 0.0%_b_ | 37.5%_a_ | 14.1% |  |  |
|  | **Grass pecking** | **Low** | 43.8%_a_ | 31.3%_a_ | 25.0%_a_ | 37.5%_a_ | 34.4% | 12.242 | 0.055 |
|  |  | **Medium** | 31.3%_a, b_ | 56.3%_b_ | 12.5%_a_ | 31.3%_a, b_ | 32.8% |  |  |
|  |  | **High** | 25.0%_a_ | 12.5%_a_ | 62.5%_b_ | 31.3%_a, b_ | 32.8% |  |  |

Each subscript letter denotes a subset of Genotype categories whose column proportions do not differ significantly from each other at the .05 level (z-test).

**Figure 1SM.** Percentage of animals exhibiting different behaviors classified as Active and Static (A), Ingestive (B), and Positive (C) or Negative (D) affects, according to genotype. Sleeping is not reported, as its average occurrence was less than 0.01%. For each behavior, bars that do not share the same letter indicate significant differences among genotypes (p<0.05); if no letters are shown, the effect of genotype was not significant.

| 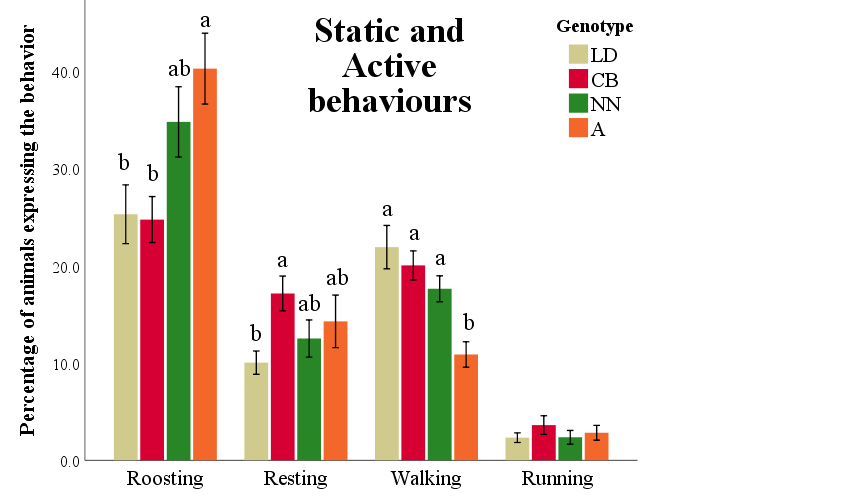A |
| --- |
| 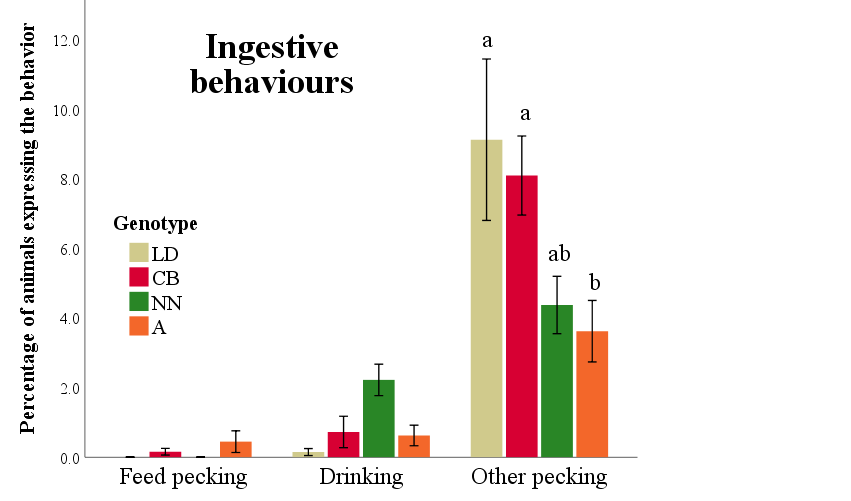B |
| 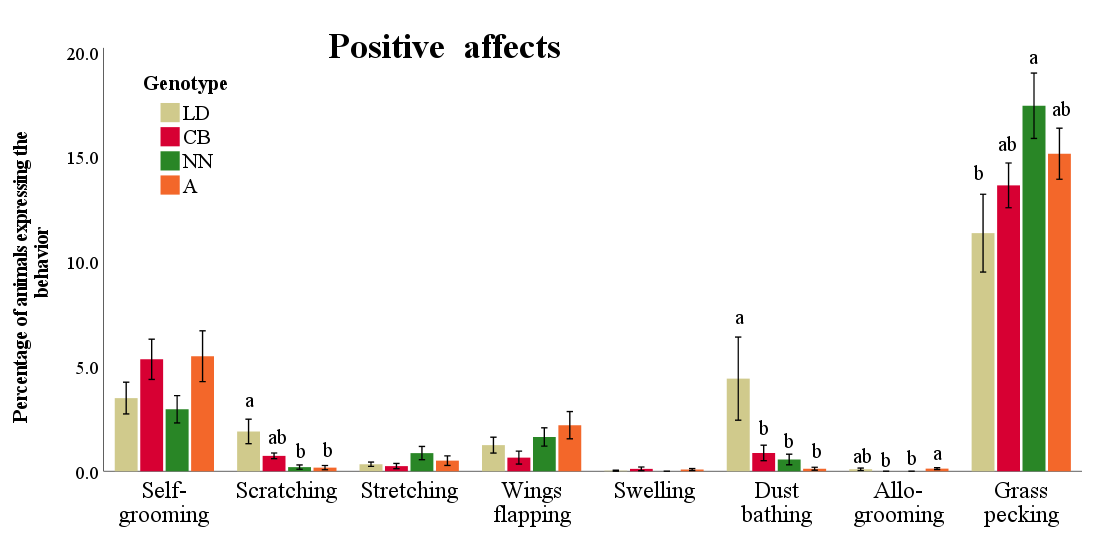  C |
| 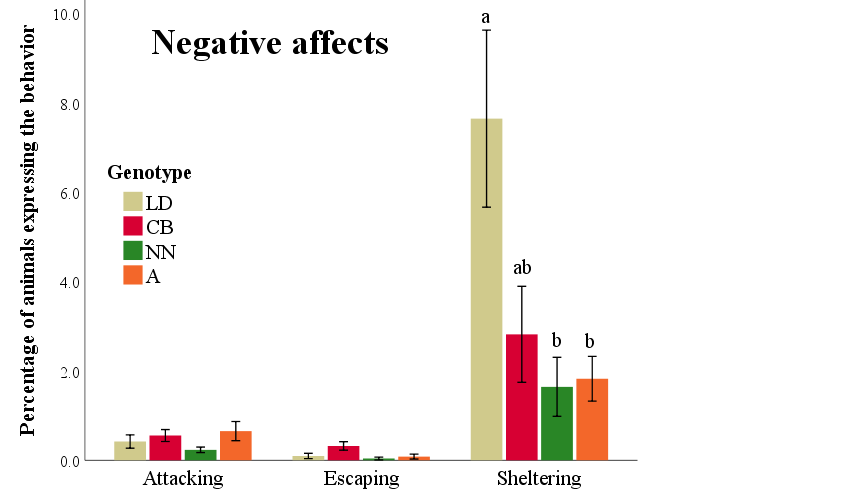D |

**Figure 2SM.** Spider graph showing all the behavioral categories (average percentages over the entire observation period) for the studied genotypes. A brief presentation of the corresponding results is also provided.


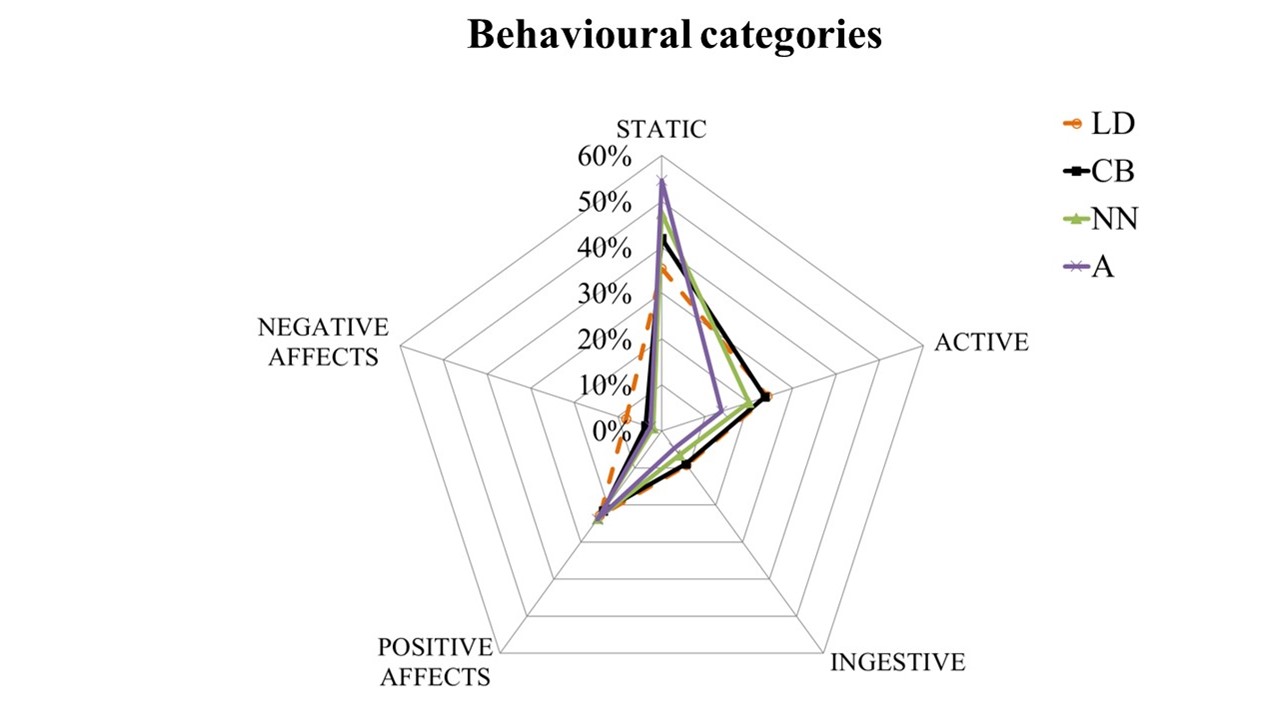


Genotype A exhibited the highest occurrence of static behaviors (Wald χ^2^ = 323.810, df = 3, *p* < 0.001), along with lower active behaviors than CB and NN (Wald χ^2^ = 35.922, df = 3, *p* < 0.01) and lower ingestive behaviors than CB (Wald χ^2^ = 10.436, df = 3, *p* = 0.008). No significant differences were observed among genotypes in positive affects (Wald χ^2^ = 2.232, df = 3, *p* = 0.526), whereas differences were detected for negative affects (Wald χ^2^ = 8.940, df = 3, *p* = 0.030). Pairwise comparisons indicated that negative affects were higher in LD than in NN (*p* = 0.041).
